# Supplementary material for: Content-rich biological network constructed by mining PubMed abstracts
Source: BMC Bioinformatics. 2004 Oct 8;5:147. doi: 10.1186/1471-2105-5-147 (PMC528731; doi:10.1186/1471-2105-5-147)
Supplement: Additional File 2 — The original results of the above study (non-essential files are deleted to keep the file size under the limit set by BMC bioinformatics). [file 1471-2105-5-147-S2.bz2 › chilibotAdditionalFile2/dip05/269ID1631061E1085/html/TPM2_JUN.html]

 


 **TPM2** and **JUN** 
  
Found 4 abstracts in PubMed, retrieved 4.  
 

 What does Google say? 
 PDF only 
| .edu only 

---

**Interactive relationship** (e.g. stimulation, inhibition, etc)

**Neutral relationship**- We show here that the expression of high molecular weight tropomyosin 2  [ **TPM2** ]  TM 2 is down regulated in c  **jun**  transformed FR3T3 rat fibroblasts.  Ref: 9690624 Cell Growth Differ, 1998

**Non-interactive relationship** (e.g. studied together, co-existance, homology, etc.)

- Down regulation of tropomyosin 2  [ **TPM2** ]  expression in c  **Jun**  transformed rat fibroblasts involves induction of a MEK1 dependent autocrine loop.  Ref: 9690624 Cell Growth Differ, 1998
